# Gunshot Wounds Made by Modern Missiles and Their Treatment1Remarks before the Medical Society of the County of Erie, June 14, 1898.

**Published:** 1898-08

**Authors:** Roswell Park

**Affiliations:** Professor of surgery, Medical Department, University of Buffalo; 510 Delaware Avenue


BUFFALO MEDICAL JOURNAL.

VOL. XXXVIII. AUGUST, 1898. NO. 1

### Original Communications.

### GUNSHOT WOUNDS MADE BY MODERN MISSILES AND THEIR TREATMENT.[1]

BY ROSWELL PARK, A. M , M D.,
Professor of surgery, Medical Department, University of Buffalo.

DESPITE the fact that half the losses entailed upon the army in a military campaign are due to death from disease, the subject of gunshot wounds will always possess for the surgeon a peculiar fascination and a large degree of humanitarian interest. The general importance of the subject, combined with its peculiar opportuneness at this time, have led me to very willingly accede to the request of our President, that I should make some general remarks upon the subject today.

Gunshot wounds began to figure in the history of surgery and surgical literature about the latter part of the XIVth. or the early part of the XVth. centuries. At this period Clemanus, of Venice, who appeared very largely in the surgery of his day, held nearly the view in which they are today regarded, *i. e.*, he did not consider them to be necessarily poisoned wounds, and treated them with warm oiled bandages, in a rude way corresponding to our modern antiseptic occlusion. Braunschweig (died 1533) took the contrary view and regarded them as poisoned wounds. Ambroise Parè (died 1590), who revolutionised the surgery of his day in more than one way, had to combat this then prevalent notion. The custom of the day was to cauterise all these wounds with boiling oil in order thus to destroy the poison. During one of the campaigns of the famous general, Monte Jan, there was such lack of this hot oil that it could not be used as he had previously seen it used. He noted at once that the wounds which had not been

1. Remarks before the Medical Society of the County of Erie, June 14, 1898.

thus cauterised did better than those in which it was used, and this clinical observation at once prompted the great reform which must ever be connected with his name. Thus by abolishing its use he went quite contrary to the teachings and practice of de Vigo (Ludovico, died 1520, to whom we owe the emplastrum de Vigo) who had been systematically cauterising them. Maggi, of Bologna, and Berengarius, of Carpi, (died 1550) also treated gunshot wounds after a very simple fashion and Wuertz, of Basle, (died 1575) bitterly censured the treatment of Gersdorff and the maltreatment of these wounds by instruments and fillets drawn through them, the use of caustics and the like. Fabricius Hildanus (died 1634) considered them to be poisoned wounds, while Purmann (died 1721) took the opposite view. The teaching of John Hunter (died 1793) was rather against their poisonous properties. And from that time on few, if any, of the great military surgeons thought much about their poisonous character.

Next to Parè the most renowned military surgeon of them all was probably Baron Larrey (died 1842), the great surgeon of the Napoleonic campaigns, the man whom Napoleon himself regarded as the "most virtuous of men." Larrey had an enormous experience and it is said that during the retreat from Moscow he made in one day 200 amputations with his own hands, a record which has never been approached by any one else. Dupuytren (died 1835) had much to say in his teachings about gunshots wounds, and Baudens (died 1857) dignified the subject by a special work entitled, Clinique des plaies d'armes à feu," published 1836. From his day down to the present, while experience accumulated, the only thing that we need particularly note in this connection is the generally prevailing impression that, while a gunshot wound was not necessarily a poisoned wound, it was practically always necessary to remove the bullet when it had lodged in the tissues; a notion which even yet prevails among those not fully informed on this subject.

It is hardly necessary at this time, nor is it included in the scope of my remarks, to contrast the horrors of the old days before the antiseptic era with the kindly results obtained now under the new method of treatment. The horrible prevalence of erysipelas, tetanus and other infections under the old regimè, the frightful mortality of the past, have not entirely disappeared as yet, but have been startlingly reduced by the new antiseptic measures. The majority of men, now, who are not necessarily fatally injured can be redeemed from the military cemeteries by the methods at present in use. But it is not necessary to digress in this direction. My purpose now is rather to invite you to the consideration of gunshot wounds as such, the mechanism of their production and perhaps in closing, a few remarks suggested by such discussion.

And first, some considerations with reference to modern small-arm projectiles. Previous to 1886 France had a 11 mm. Chassepot weapon. In 1886 the caliber was reduced to 8 mm. At about the same time various other nations reduced also the caliber of their standard weapons from 10.75 mm. or 12.50 mm. down to the 8 mm. size of France, Austria and Denmark. Chili and Peru use now a 7.6 mm. bullet; China and Japan, 7.9 mm. In the recent Chitral campaign the British have been using a 7.9 mm. bullet in their Lee-Mitford rifles, while the Italians in their last Abyssinian campaign used a 6.5 mm. ball. To this last fact, indeed, as I shall allude a little latter, has been ascribed part of the misfortune which the Italians suffered in that campaign.

Now contrast these with the projectiles used in our civil war, when the bullets used were .50″ to 58″, or 12.5 to 14.5 mm. in diameter, also with the rifles until recently in use in our state service, which had a .45″ caliber. The rifle which our regular infantry and cavalry now use is the Krag-Jorgenson arm, and has a caliber of .30″, equal to 7.62 mm. The Mauser ball, now used by the Spanish, is a little smaller than this, *i. e.*, 7 mm. The marines in our navy use a still smaller caliber, .236″, or 6 mm. Our infantry bullet has a length of 4 calibers, weighs 220 grains and is jacketed with German silver. The rifle barrel is so grooved that the ball makes one rotation for every 9 inches of flight, and it leaves the muzzle with a velocity of over 2,000 feet to the inch. The marked increase in both velocity and rapidity of rotation over the old projectiles is due to this reduction of caliber.

This matter of rotation on its axis of the bullet in its flight is one of no small interest to military surgeons since the so-called "explosive effect" of the projectile is in large degree due to it. When the bullet leaves the gun barrel it is probably like a heavy top which has just been spun, and while revolving on its own axis makes quite an excursion, or as the boys would say "wobbles" a good deal. After the bullet has gone a certain distance, perhaps 200 or 300 yards, it settles down into a more steady motion, and, then, after 1,200 or 1,500 yards losing its velocity, begins to wobble again. It is while the ball has this extreme of added complex motion that it is capable of producing a most disastrous lateral or explosive effect. Such, at least, is the theoretical explanation which has been offered, and which has much to justify it. These explosive effects began to be noted when the French reduced the diameter of their Chassepot balls. Indeed, so destructive were they that the French were at first accused of using explosive bullets, a form of missile prohibited in warfare between civilised nations.

The effect of jacketing or mantling bullets is to make a much lighter ball carry very much farther and to give it a very much enhanced penetrating effect. If the mantle be undisturbed, such a bullet thrown from one of our rifles will carry a distance of some three miles, if given a proper elevation ; but when the jacket is loosened in its passage through the gun barrel or through some firm tissue, like bone, it is capable of inflicting a very ragged and destructive wound. During the campaigns in India and Abyssinia some of the British soldiers had a habit of loosening the end of the mantle before the cartridge was fired, so that the bullet, though not carrying so far, was capable of inflicting a most serious wound. Balls so treated were known among the soldiers as " dum-dum " bullets.

Every gunshot wound is a resultant of the penetrative and the lateral forces exerted by the projectile. In proportion to the resistance offered the former is transformed into the latter. If the initial force or energy of the ball be completely expended in penetration it will leave a clean track through the tissue, but if otherwise not. The penetrative effect of a projectile is due (1) to its hardness, (2) to its velocity, (3) to its size—the smaller the more rapid, (4) to its weight, (5) to the softness of the tissue, and (6) to the fluidity of contents of the part wounded. In the matter of its penetration, as already stated, much depends upon the jacket or mantle. The steel-covered ball is much more penetrative than that covered with German silver. For instance, in a given experiment a .30″ bullet mantled with German silver penetrated 5.3″ of oak, while the same sized ball fired from the same weapon, but covered with steel, penetrated 19.5″ of oak. It is an established law that resistance increases faster than does the penetrability of a given material due to the velocity of the ball, *i. e.*, beyond a certain point additional velocity causes only lateral destruction.

The lateral, or so-called explosive effect of the bullet is due (1) to the softness of its material (2) its velocity (3) its size or frontage (4) its weight (5) rapidity of rotation and (6) hardness of the tissues. These are all self-evident or axiomatic statements, which probably need no further definition.

Let us consider next the hydrodynamic properties of fluids and soft tissues. The incompressibility of fluids is everywhere understood, but this fact is not generally known to be an explanation of the explosive effect of modern bullets upon tissues and hollow viscera containing fluids, as, for example, the brain, the heart, the stomach, the intestine and even the liver and spleen. So complete are the disrupting and disorganising effects of these missiles upon these tissues that several inches of bowel may be completely shattered by a perforating ball, and that at 1,100 meters even, or more, the brain may be completely pulpified. In certian other tissues, especially the muscles, the small balls will completely pass through the part without injuring it, as no large bullet could. This is particularly true of our small revolver bullets. Hunters take advantage of these known facts and wishing, of course, to disable such game as they may hit, prefer a soft large bullet to a small hard one. Hugier, in 1848, was perhaps the first to call attention to this explosive effect of balls in soft organs, and to give them their proper hydrodynamic explanation. This has since been quite fully demonstrated by Kocher, in Germany, Horsley, in England, and Woodhull of our own army medical corps.

There have prevailed most opposite opinions in recent years as to the humanity or propriety of using small bullets in modern weapons; some holding them to be the least fatal and most humane, others taking an exactly opposite view. These wide variations of opinion are due to the widely variant effects of small caliber projectiles, which can rarely be predicated or determined in advance, and which must depend in considerable degree upon the physical condition of the tissues wounded, of the velocity of the ball, of the distance from the weapon and the consequent excursion of rotation of the bullet. Moreover, so-called "vibratory physics," to which much attention is now being paid, are capable of shedding considerable light upon the subject, for much of the true explanation is undoubtedly included in complex vibrations into which the bullet itself is thrown, and which it transmits to the tissues. If a bullet in its flight can throw the air through which it passes into such vibration that there is transmitted to the ear of the bystander the peculiar whistling or "ping" of the ball, it is possitive that the tissues and fluids, through which it passes in inflicting a wound, must be thrown into much more positive and destructive sympathetic vibrations. And so it happens that sometimes such a bullet will make a clean hole through the bone and at other times completely shatter it. Woodhull has lately called attention to this fact and shown how it depends upon what may be known as the nodal point of the bone. Take this illustration which will be familiar to most of us: We all remember how with a baseball bat in our hands we may sometimes strike the ball in such a way that a most disagreeable tingling or benumbing sensation is transmitted to our hands, while at other times there will be no unpleasant sensation, even though the ball be struck with considerable force. This all depends upon nodal points in the bat—in other words, on just what point of the stick comes in contact with the ball. And so it may happen that a bullet striking the upper end of the tibia may pass through it with little or no splintering, while striking its lower end it may completely shatter its upper extremity and so disorganise everything as to necessitate an amputation above the knee ; an event which has actually happened in practice.

As is always the case with a solid body moving through fluid a certain amount of wave motion is set up. Whether the solid body move slowly or rapidly it displaces fluid, which must make room for it and then must readjust itself. When to motion in a straight line there is added a great centrifugal velocity and the effect of rotation, the effect upon the fluid and its container may be extremely explosive. There must also be taken into consideration here what the physicists call " cavitation " or the effect of the instantaneous vacuum which is formed behind a bullet in its rapid flight. This is something which may be noticed in a mild degree if one stand on the rear platform of a rapidly moving train. There is invitation, then, to the production of a vacuum, as may be noticed by the tendency to suck in dust and small fragments of any kind. Naval constructors notice the same phenomenon also in the rear of a screw propeller, since the water which is sucked in behind the moving vessel gets to be, in course of time, that is when the motion becomes too rapid, a positive obstacle to speed beyond a certain degree. Now there is cavitation behind every bullet and the consequence thereof is a series of wave-like rebounds, sufficient even to draw in and bulge out the flexible bone of a living skull. Under these circumstances, therefore, the wound of exit may be very large.

These effects can be most easily demonstrated, as Woodhull as shown, by firing rifle balls through a series of ordinary round tin cans more or less filled with fluid and lying upon the side. With an empty can the ball will pass through top and bottom making cleanly punched holes, but in proportion to the amount of fluid present and its density we get explosive effects by which, for instance, the bottom of the can will be completely torn loose, or the entire can practically exploded. The effect upon the heart, then, of such a bullet will depend in considerable degree on just how much blood the heart may happen to contain at the instant of injury, it being known that sometimes the heart is so exploded, when thus wounded, as to be simply unrecognisable.

In view, then, of what I have stated, you will regard it as questionable whether the new missile is humane or not. Demosthène held it to be more destructive than the old bullet, a view in which Lt. Stiles, Maj. Halley and Dr. Griffith concur. Girard also considers its humane effects to be purely mythical. The genius of modern war is, if possible, to prevent the enemy from fighting by injuring him or disabling him, and only to kill him if necessary. Obviously, with this new bullet the only way to absolutely stop him is to kill him promptly, and perhaps barbarously, for unless we kill him he may continue to advance and kill us. This sort of thing has repeatedly occurred in the campaigns in India and Abyssinia. During the Italian war there were cases of perforating chest wound which suffered only slight shock and did not disable, while the patient quickly recovered. The same was true also in the recent Chinese war. To stop the enemy or disable him at long range is the ideal of modern fighting, but it requires an accuracy of markmanship which can not be obtained with the average soldier, and is only obtainable with long trained sharp-shooters. All these things considered, then, there are many scholars in the art of modern warfare who are now contending for a return to large caliber weapons, at least for the main body of the army. Large soft bullets are more quickly stopped, give up all their energy, and deliver a far more serious actual blow than do the new small weapons. It is a fact that the Italians were defeated in Abyssinia, a short time ago, largely because of the failure of their 6.5 mm. Mannlicher bullets to disable the enemy. So marked was this failure that for some time their ammunition was supposed to have been tampered with, so ineffective was it. It has been stated as a great argument in favor of our new projectile that it is capable of passing through 12 to 15 men at ordinary short range. This is true providing it does not strike fluid tissues, but it loses energy by exploding fluid-containing organs, like the heart, brain, bladder, and the like, when it will lose energy so fast that it cannot pass through more than three or four men at most (Woodhull, *New York Med. Jour.*, April 30, 1898). In the Chitral campaign the prisoners accounted for the absence of dead enemies on the battle-field by saying that unless a man was shot through the head or bowels he did not die, and that he was not even disabled unless by a wound of his lower joints. But the Chitrals themselves, with their .45″ rifles disabled a great many of the British soldiers and killed almost every time they hit.

Study now for a moment the effects of the new ball on particular tissues. In the skin, under ordinary circumstances, there will be little or no difference between the wounds of entrance and of exit. If such a ball pass through a blood-vessel of the larger class the edges of the wound will be so clean-cut that no retraction of the inner coat can take place ; consequently, no spontaneous cessation of bleeding can occur. In other words, a wound of a large vessel will be quickly fatal unless bleeding be promptly checked. In the muscles the explosive action of these bullets will be least marked, unless the bone be also shattered. In the bones the explosive effects will be most marked at short range, for reasons which I have already alluded to. Beyond about 400 yards range they will be less so, while, again, at long range there will ordinarily be less shattering and comminution than the old balls used to produce, and a clean penetration will be the rule unless the nodal point is struck. In the joints themselves the small bullet rarely makes a severe injury save by shattering the bone. In the skull up to 1,200 to 1,500 yards complete shattering of the bone and disruption of the brain will take place. At short ranges the destruction will be even more terrific and the head so struck will lose all resemblance to human skull. In the chest, clean perforation of the lungs will ordinarily take place, unless pieces of bone are driven in. What may occur in the heart has been already alluded to. Tendons, unless struck squarely, are pushed aside by the bullet. The abdomen may be perforated even at the longest effective range of a weapon and multiple perforations of the intestines, with often complete destruction, may take place. The soft organs, like the liver, spleen and even the kidney may become pulpified.

Thus it will be seen that the nonfatal wounds of the future are likely to be less severe and to heal more rapidly and with fewer complications, also to require less radical treatment than those produced by the old bullets. That is, gunshot wounds inflicted by the new weapons will largely resolve themselves into two classes—the horribly destructive and the less severe (Davis, Annals of Surgery, Vol. XXV., p. 36.)

There has been much conjecture as to the effect of the heat of discharge upon the sterile or nonsterile condition of the bullet ; it having been generally supposed that this heat was sufficient to completely sterilise the bullet by the time it leaves the muzzle. Captain Legard, of our army medical corps, made sometime ago a series of careful experiments with a view to the determination of this matter. He dipped the front ends of the cartridge in various cultures of bacteria, using largely anthrax for this purpose, and then fired them into the various materials from which cultures or inoculations were made. He found that the heat was not great enough to sterilise bullets contaminated with anthrax, though it might be enough to kill less resistant organisms. In a general way, the results of his experiments went to show that this heat could not be relied upon for purposes of such sterilisation.

Without taking further time to consider the effect of modern missiles, let me now invite your consideration to a few remarks upon the treatment of the injuries which they inflict. For a number of years military and experienced civil surgeons have accepted the dictum of Esmarch, which may be summed up in these words, "when the ball has ceased to move it has ceased to do harm." In other words, the harm which a bullet does is done in its flight; so as soon as it has come to rest it is an innocent foreign body. It has taken many years of experience to educate men away from the ideas that a ball is necessarily a poisonous substance, and that the very first indication is for its removal. This is a mistake which in time past wrought incalculable harm. The first indication in every wound of this character is the checking of hemorrhage, the second, so far as the surgery of the battle-field is concerned, is primary antiseptic occlusion, or if you like to put it in other terms, abstention from interference. I need not remind many of the men in this room that during our civil war, for instance, a wound of the ankle-joint meant usually an amputation of the leg, a wound of the knee-joint an amputation of the thigh and a wound of the hip-joint an amputation at that joint, which was almost inevitably fatal. Contrast such results as those under the old regimé with the new method of treatment by noninterference The valuable lesson of such masterly inactivity was conspicuously taught us for the first time in the campaign in the Caucasus during the Turko-Russian war. At this time the Russian field-surgeon, Reyher, made an astonishing record by demonstrating that by primary antiseptic occlusion of gunshot wounds of joints, *i. e.*, by the let-alone policy, he not only saved most of the limbs thus injured, but got more or less useful and functionating joints. No more convincing demonstration of the value of antiseptic methods was ever offered than this, which revolutionised modern military surgery. Of course, you will not understand me as meaning that no bullet is ever to be removed, and no wound ever to be treated in any other way. What I mean is that primary abstentation from everything save checking of hemorrhage and occlusion or hermetic sealing of the wound is the proper emergency dressing.

What I specially want to condemn is the indiscriminate, thoughtless and usually injurious use of the probe. There is no lesson which it may teach upon the battle-field which cannot be learned without it, while the objections to its use are many. It is rarely, if ever, clean; it stirs the blood clot and tends to reproduce hemorrhage; it permits access of air to the deeper parts and it rarely gives information which is of any real service at the time. In the hands of most men it is a dangerous instrument and it would be well if we left it out of the ordinary pocket case, or military surgeon's outfit. It is an instrument to be used, if at all, in a division hospital and not at the front. Even should the bullet be touched with it the surgeon who uses it rarely has at hand the means for removal of the ball when he finds it. In civil practice I have seen great harm come from its use; how much more, then, may happen in military work; If an important cavity be entered by the ball that fact can be appreciated without the probe; if a large vessel be divided, probing the wound will only stir up fresh blood. If nerve trunks have been cut across, the probe is not needed for detection of the fact and four times out of five the ball has come to rest in a place which cannot be touched by the probe, even were there are no other objection to its use.

On the other hand, in a division hospital, or at some suitable place where deliberate work can be done, it is well to be provided with a telephone probe and a Röntgen apparatus, by which bullets or other foreign bodies can be accurately located and removed under every possible aseptic precaution, when this is considered wise or necessary.

Considering that our soldiers are now for the most part provided with packages of dressings with which to render their own first aid and that they are pretty generally tought the advantages of primary antiseptic occlusion, we can feel reasonably confident that the best thing will now be done in our armies and under the stress of military combat. I have repeatedly tried to secure the introduction of this dressing packet in the police force of this and other cities and the instruction of the men in its ordinary use, but have for the most part not succeeded. I need not remind you how advantageous such a course would be—for instance, in our own city.

In conclusion I would only say that the rules of military surgery are the rules which for the most part are necessary deductions from the teachings of the civil surgeons, and that they are the simplest of all formulæ which can be carried out to meet emergencies. By their judicious practice many more lives will be saved and we will hear much less of sepsis, erysipelas, hospital gangrene and tetanus than ever before in our campaigns and will have warded off the greatest dangers which have in time past threatened our brave soldiers and devastated our military hospitals.

510 DELAWARE AVENUE.

